# Trifluridine/tipiracil as a therapeutic option in real life setting of metastatic colorectal cancer: An efficacy and safety analysis

**DOI:** 10.3389/fphar.2022.1041927

**Published:** 2022-10-13

**Authors:** Daniel Sur, Cristina Lungulescu, Ștefan Spînu, Alecsandra Gorzo, Elena-Adriana Dumitrescu, Dan Ionut Gheonea, Cristian-Virgil Lungulescu

**Affiliations:** ^1^ Department of Medical Oncology, The Oncology Institute, Cluj-Napoca, Romania; ^2^ Department of Medical Oncology, University of Medicine and Pharmacy, Cluj-Napoca, Romania; ^3^ Doctoral School, University of Medicine and Pharmacy of Craiova, Craiova, Romania; ^4^ Institute of Oncology, Bucharest, Romania; ^5^ Gastroenterology Department, University of Medicine and Pharmacy of Craiova, Craiova, Romania; ^6^ Oncology Department, University of Medicine and Pharmacy of Craiova, Craiova, Romania

**Keywords:** metastatic colorectal cancer, trifluridine/tipiracil, Romanian population, toxicity analysis, real-world data

## Abstract

**Background:** In the phase III RECOURSE trial, the orally administered combination trifluridine/tipiracil (FTD/TPI) demonstrated a survival benefit and an acceptable safety profile, earning approval as a third-line therapy in metastatic colorectal cancer (mCRC). This study aimed to assess the efficacy and safety of FTD/TPI in daily clinical practice in Romanian population.

**Methods:** A single-center, retrospective, and observational study analyzed patients with mCRC that received chemotherapy with trifluridine/tipiracil between May 2019 and May 2022 at the Oncology Institute Prof. Dr. Ion Chiricuță in Cluj-Napoca, Romania. Study endpoints included safety, and median progression-free survival (PFS).

**Results:** In this Romanian cohort (*n* = 50) the most common treatment-emergent adverse event was haematological toxicity (76%): anemia (50%), leucopenia (38%), neutropenia (34%), and thrombocytopenia (30%), followed by fatigue (60%), and abdominal pain (18%). Overall, the median progression-free survival was 3.85 months (95% CI: 3.1–4.6 months). PFS was significantly correlated with the number of FTD/TPI administrations and prior surgery.

**Conclusion**: Our study corroborated the previously described safety profile for FTD/TPI in the third-line setting, and demonstrated relatively superior mPFS.

## Introduction

Colorectal cancer (CRC) is one of the most frequent malignancies and leading causes of cancer-related mortality worldwide (Ferlay et al., 2015; Favoriti et al., 2016). Although overall survival (OS) has improved, there are few regimens available for patients who progress beyond first- and second-line treatment (Vogel et al., 2017; Arnold et al., 2018).

Fluoropyrimidines have been generally regarded an essential component of colorectal cancer treatment (Meyerhardt & Mayer, 2005). These agents predominantly inhibit thymidylate synthase, an enzyme involved in pyrimidine nucleotide synthesis. The ability of fluorouracil (5-FU) to bind to thymidylate synthase has been improved by combining it with folinic acid (Sobrero et al., 2000). The current standard of care for mCRC includes the addition of oxaliplatin (FOLFOX) or irinotecan (FOLFIRI) to fluorouracil and folinic acid, along with a vascular endothelial growth factor (VEGF) inhibitor (e.g., bevacizumab) or an epidermal growth factor receptor (EGFR) inhibitor for RAS wild-type tumors (e.g., cetuximab or panitumumab) (Yoshino et al., 2018). Trifluridine (FTD) was developed nearly 50 years ago, close to the introduction of fluorouracil, and demonstrated antitumoral activity (Heidelberger & Anderson, 1964; Heidelberger et al., 1965; Dexter et al., 1972). However, subsequent drug development was terminated because the required dosing schedule for trifluridine exhibited a toxicity profile that was unacceptable for long-term use (Dexter et al., 1972). It was not until approximately 15 years ago that tipiracil (TPI) hydrochloride, which inhibits the fast degradation of trifluridine and enables the preservation of acceptable plasma concentrations of the active medication, was developed (Fukushima et al., 2000).

The subsequent combination of trifluridine and tipiracil (FTD/TPI) to develop TAS-102 prompted the preclinical and clinical trials that led to its approval for refractory mCRC in Japan in March 2014 (Yoshino et al., 2016). Firstly, a Japanese phase II study (JapicCTI-090880) established the safety and efficacy of TAS-102 monotherapy in patients with refractory mCRC (Yoshino et al., 2012). Following that, the RECOURSE trial (Mayer et al., 2015) was successful in gaining authorization in both the United States and Europe in September 2015, and April 2016, respectively (Mulet et al., 2018). According to the phase III study (NCT01607957), FTD/TPI increased both median progression-free survival (mPFS) and median over-all survival (mOS) when compared to placebo, from 1.7 to 2.0 months and 5.3–7.1 months, respectively (Mayer et al., 2015).

These findings were corroborated by a second phase III trial (TERRA) conducted in an all-Asian demographic (Xu et al., 2018). In light of the promising outcomes of the clinical studies, an international phase IIIb research, PRECONNECT (NCT03306394), was launched to further analyze FTD/TPI in a sizable cohort of patients engaged in normal clinical practice (Bachet et al., 2020).

Post hoc analyses of the PRECONNECT research are being conducted on a country-specific basis due to disparities in disease treatment between states, with publications so far available for Italy (Zaniboni et al., 2021), and Turkey (Ozet et al., 2022).

In February 2017, the National Oncology Program of Romania covered trifluridine/tipiracil for patients with mCRC who had previously had two or more lines of treatment or who were ineligible for intense chemotherapy.

To the best of our knowledge, this is the first study to document real-world experience of using FTD/TPI in mCRC in Romania.

## Materials and methods

### Study design

The present investigation is non-interventional, retrospective, single-center study that analyzed patients with metastatic colorectal cancer that received chemotherapy with trifluridine/tipiracil between May 2019 and May 2022 at the Oncology Institute Prof. Dr. Ion Chiricuță in Cluj-Napoca, Romania. The study was conducted in compliance with the principles of the Declaration of Helsinki, and all participants provided written, informed consent.

### Patients

Patients included in the study had to be at least 18 years old, have a biopsy-confirmed adenocarcinoma of the colon or rectum with metastatic lesions, and have an Eastern Cooperative Oncology Group performance status (ECOG-PS) of 0–2. Patients were required to have undergone a minimum of two prior regimens of standard chemotherapy consisting of fluoropyrimidine, oxaliplatin, and irinotecan (which included adjuvant setting if recurrence happened within 6 months), and bevacizumab, or anti-epidermal growth factor receptor (EGFR) monoclonal antibody for RAS-wild-type tumors.

Baseline information such as demographic data, ECOG PS, disease characteristics, RAS-mutation status, treatment description (prior systemic regimens and surgeries), number of trifluridine/tipiracil cycles, toxicities, disease response, and date of progression were collected from patients’ medical records.

### Treatment

Trifluridine/tipiracil was given orally twice daily at a dosage of 35 mg/m2, in a 28-day cycle that included five treatment days and 2 rest days for 2 weeks, followed by a 14-day rest period. This completed one treatment cycle, which was repeated every 4 weeks. Treatment continued until disease progression, unacceptable toxicity, or withdrawal of consent.

### Outcomes

Study endpoints included safety, and median progression-free survival (PFS). PFS was defined as the time elapsed between the beginning of trifluridine/tipiracil treatment and the first recorded disease progression or death from any cause. Treating physicians determined the intervals at which tumor response was measured using RECIST 1.1. The National Cancer Institute Common Terminology Criteria for Adverse Events (NCI CTCAE), version 4.0, was used to grade all toxicities.

## Statistical analysis

All the data was collected in an Excel worksheet and analyzed using GraphPad 9.4.1 (GraphPad Software, San Diego, CA, United States). Continuous variables were presented as mean ± standard deviation, and categorical variables as number (percentages). The Kaplan-Meier method was used to estimate the curve corresponding to the progression-free survival (PFS). The patients alive at the time of last follow-up were censored. Cox regression was performed for PFS with the main known prognostic factors: age, gender, tumor sidedness, RAS mutation, lymph node involvement, surgery, number of metastatic locations, number of FTD/TPI administrations. The threshold for statistical significance was 5%.

## Results

A total of 50 patients with metastatic colorectal cancer were included in the study. Baseline characteristics of enrolled patients and reported toxicities are summarized in Table 1. There were 26 male and 24 female patients with a median age of 65.5 (range, 31–86) years. Young (≤45 years) and elderly (≥70 years) patients represented 16% and 38% of our sample, respectively. The bulk of study participants had an ECOG performance level of 1%–40%, followed by ECOG 2 (36%), ECOG 3 (14%), and ECOG 0 (10%). Studying the comorbidities (60% patients had comorbidities), most of them had heart disease (30%) and hypertension (18%). Left-sided tumors were the most common (74%) presenting with cancers in the rectum (36%). Of 50 patients, 19 (38%) had wild type RAS status and 19 (38%) were mutated. Lymph node involvement was observed in 90% of patients, among which 8 (16%) were N0, 17 (34%) were N1 and 20 (40%) were N2. The liver was the principal common metastatic site (78%), followed by lung (56%), peritoneal (36%), nymph nodes (22%), bone (16%) or brain (4%). Most of the patients had more than 3 metastatic organ locations (44%). Surgery was performed for most of the patients (74%) (Table 1).

**TABLE 1:** Baseline patients’ characteristics.

Characteristics	Patients given FTD/TPI (*n* = 50)
Age (mean ± SD)	62.9 ± 12.9
≤45	8 (16%)
46–69	23 (46%)
≥70	19 (38%)
Gender	26 (52%)
Male	24 (48%)
Female	
Comorbidities	
Yes	30 (60%)
No	8 (16%)
N/A	12 (24%)
Type of comorbidities	
Hypertension	9 (18%)
Heart disease	15 (30%)
Smoking/alcohol	0
Diabetes	2 (4%)
Others	25 (50%)
Primary tumor site	
Left	37 (74%)
Right	8 (16%)
Rectum	18 (36%)
RAS status	
Wild-type	19 (38%)
Mutated	19 (38%)
N/A	12 (24%)
Lymph node involvement	
Yes	45 (90%)
No	0
Missing data	5 (10%)
Lymph node involvement	
N0	8 (16%)
N1	17 (34%)
N2	20 (40%)
No	0
Missing data	5 (10%)
Location of metastases	
Peritoneal	18 (36%)
Liver	39 (78%)
Lung	28 (56%)
Lymph nodes	11 (22%)
Bone	8 (16%)
Brain	2 (4%)
Others	3 (6%)
Number of metastatic organ locations	
1	10 (20%)
2	18 (36%)
≥3	22 (44%)
Surgery	
No surgery delivered	23 (26%)
Yes	37 (74%)
First-line chemotherapy regimens	
Oxaliplatin-based chemotherapy + Bevacizumab/cetuximab	33 (66%)
Irinotecan-based chemotherapy + Bevacizumab/cetuximab	7 (14%)
Second-line chemotherapy regimens	
Oxaliplatin-based chemotherapy + Bevacizumab/cetuximab	1 (2%)
Irinotecan-based chemotherapy + Bevacizumab/cetuximab	24 (48%)
Number of FTD/TPI administrations in 3rd line	
Mean ± SD	4.58 ± 3.91
≤5	25 (80%)
6–9	3 (10%)
≥10	3 (10%)
Missing data	—
Number of FTD/TPI administrations in 4th line	
Mean ± SD	4.0 ± 2.1
≤5	10 (83%)
6–9	2 (17%)
≥10	0
Missing data	—
Post-FTD/TPI adverse events	
Yes	40 (80%)
No	3 (6%)
N/A	7 (14%)
ECOG Status	
0	5 (10%)
1	20 (40%)
2	18 (36%)
3	7 (14%)

Data are presents as frequency (percentage) or mean (±SD).

Regarding first-line, oxaliplatin-based chemotherapy combined with bevacizumab or cetuximab was the dominant choice (66%), whereas irinotecan-based chemotherapy combined with bevacizumab or cetuximab was chosen for 14% cases.

Recorded post-FTD/TPI toxicities were registered in 80% of the study population (Table 2). The most common adverse event was low blood count (76%), followed by fatigue (60%), anemia (50%), leucopenia (38%), neutropenia (34%), thrombocytopenia (30%) and abdominal pain (18%). Interestingly, when grade 3 AEs are considered–respecting CTCAE v5.0 grading, the majority of patients (24%) encountered grade 3 neutropenia, followed by grade 3 leucopenia (18%), grade 3 anemia (4%) and only one patient experienced Grade 3 fatigue (2%).

**TABLE 2:** Reported toxicities (CTCAE).

Reported toxicities	Patients given FTD/TPI (*n* = 50)
Any Grade AE	
Low blood count	38 (76%)
Leucopenia	19 (38%)
Anemia	25 (50%)
Thrombocytopenia	15 (30%)
Neutropenia	17 (34%)
Tiredness (fatigue/weakness)	30 (60%)
Nausea	4 (8%)
Vomiting	2 (4%)
Decreased appetite	6 (12%)
Diarrhea	2 (4%)
Abdominal pain	9 (18%)
Fever	1 (2%)
Grade 3 AEs	
Leucopenia	9 (18%)
Anemia	2 (4%)
Neutropenia	12 (24%)
Tiredness (fatigue/weakness)	1 (2%)

Data are presents as frequency (percentage).

**FIGURE 1:**
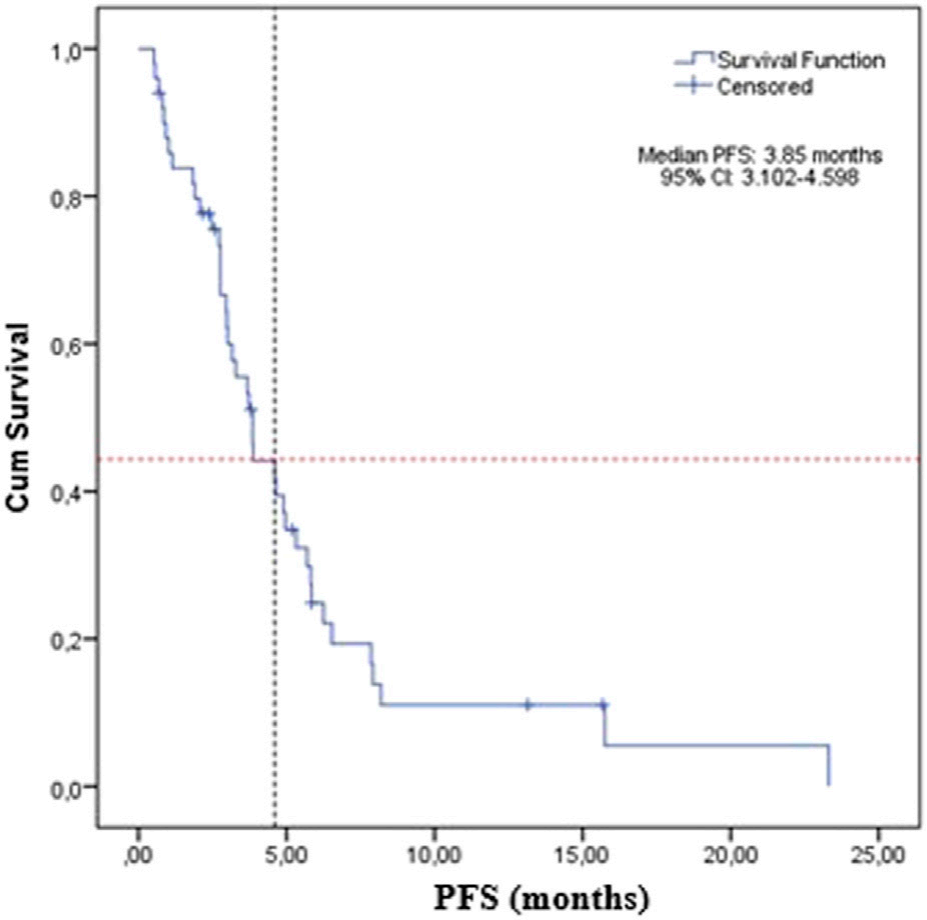
Progression-free survival of the sample treated with FTD/TPI.

The median PFS was 3.85 months (95% CI: 3.1–4.6) for the whole study group (Figure 1).

**FIGURE 2:**
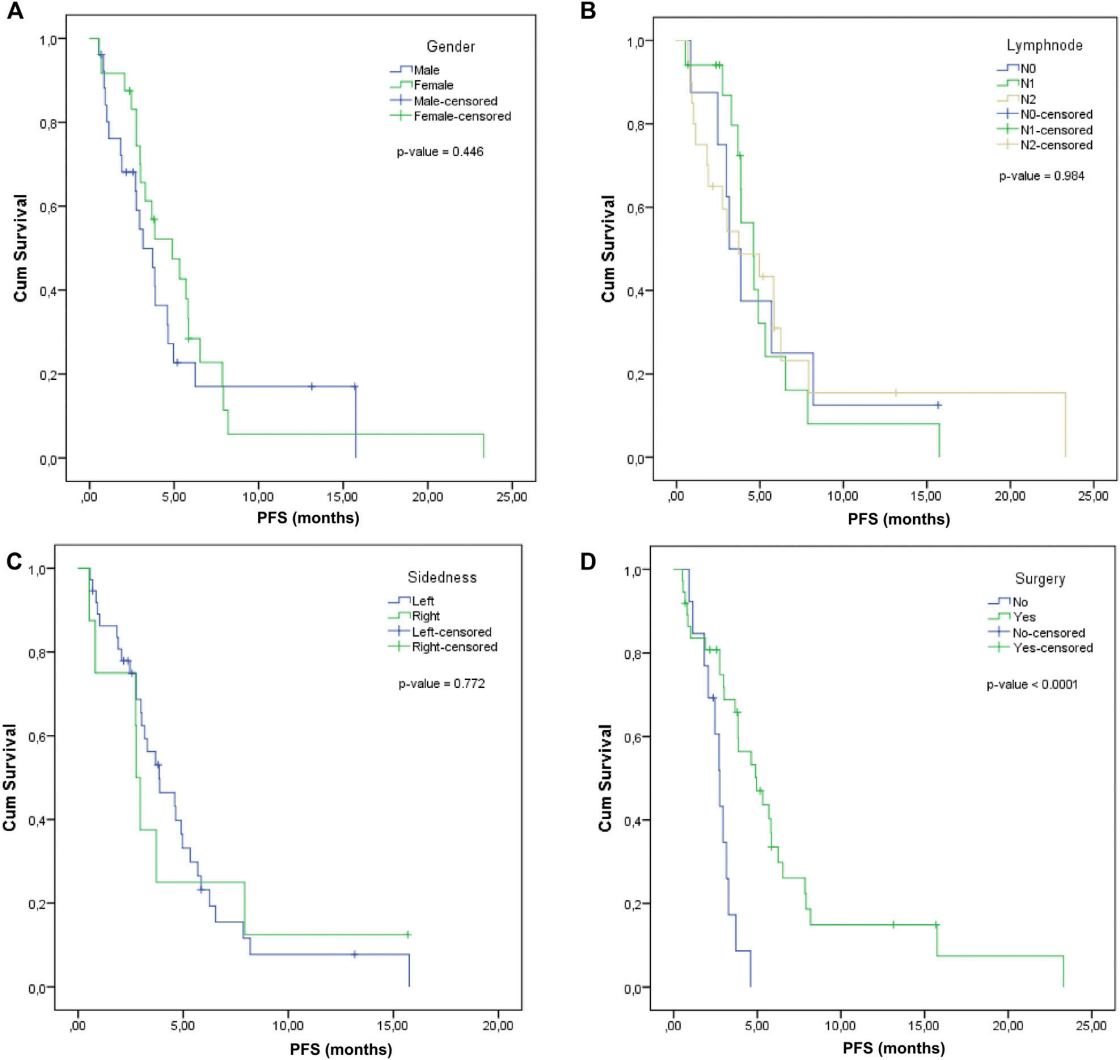
Progression-free survival according to **(A)**, Gender, **(B)**, Lymph node involvement, **(C)**, Tumor sidedness, **(D)**, Surgery.

For male patients with metastatic colorectal cancer, the median PFS was 3.16 months (95% CI: 1.94–4.38) and for female patients, the median PFS increased to 4.9 months (95% CI: 2.5–7.3), but the differences were not significant (*p*-value = 0.446) (Figure 2A).

The median PFS was calculated according to the lymph nodal status: for N0, it was 3.16 months (95% CI: 0.97–4.35), for N1, it was 4.6 months (95% CI: 3.32–5.88), and for N2, it was 3.88 months (95% CI: 2.79–4.97). The comparative difference between them was not statistically significant (*p*-value = 0.984) (Figure 2B).

Log Rank comparison on sidedness showed no statistically significant differences between the left and right: 3.85 months (2.14–5.56) vs. 2.76 months (2.44–3.08), *p*-value = 0.772 (Figure 2C).

A significantly superior median PFS was observed in the case of patients that previously received surgery (4.96, 95% CI: 3.03–6.89) compared to those without surgery (2.76, 95% CI: 2.29–3.23), with log rank *p*-value < 0.0001 (Figure 2D).

The potential predictors of PFS were investigated based on Kaplan-Meier, univariate and multivariate Cox regression and only number of FTD/TPI administrations and surgery were significantly associated with PFS (Table 3). We confirmed the prognostic value of number of FTD/TPI administrations (*p*-value < 0.0001) and surgery (*p*-value < 0.0001) on PFS. On univariate Cox regression analysis, patients with less than five doses had significantly inferior median PFS (3.16 moths vs. 7.92 months), HR = 0.18 (95% CI: 0.07–0.51), *p*-value < 0.0001. Moreover, previous surgery was also associated with PFS (log rank *p*-value < 0.0001): the impact of previous surgery was associated with a superior PFS compared to patients who did not benefit from any surgery. Notably, these observations were confirmed on multivariate analysis after adjustment for covariates.

**TABLE 3:** Association of baseline characteristics with PFS.

Characteristics	Kaplan-meier survival analysis with log-rank test		Univariate cox regression analysis		Multivariate cox regression analysis	
	PFS (95%CI)	*p*-value	HR (95%CI)	p-value	HR (95%CI)	p-value
Gender (female vs. male)	3.85 (3.1–4.6)	0.446	1.27 (0.68–2.38)	0.449	—	—
Age (<50 years vs. ≥ 50 years)	3.85 (3.1–4.6)	0.949	0.97 (0.43–2.21)	0.950	—	—
Comorbid conditions (yes vs. no)	3.68 (2.5–4.9)	0.844	1.09 (0.46–2.58)	0.845	—	—
Tumor sidedness (left-sided vs. right-sided)	3.68 (2.7–4.7)	0.772	1.13 (0.49–2.61)	0.775	—	—
Lymph node involvement (N2 vs. N0/N1)	3.88 (2.8–4.97)	0.858	0.94 (0.48–1.85)	0.858	—	—
Number of metastatic organ locations (<3 vs. ≥3)	3.85 (3.1–4.6)	0.759	1.1 (0.6–2.1)	0.760	—	—
Number of FTD/TPI administrations (≤5 vs. >5)	3.85 (3.1–4.6)	<0.0001	0.18 (0.07–0.51)	0.001	0.21 (0.07–0.59)	0.003
Surgery (no vs. yes)	3.85 (3.1–4.6)	<0.0001	0.26 (0.12–0.58)	0.001	0.34 (0.15–0.76)	0.008

## Discussion

FTD/TPI is an anti-tumor medication administered orally that consists of trifluridine (a nucleoside analogue), and tipiracil (a thymidine phosphorylase inhibitor), and is registered for mCRC refractory to standard regimens in over 93 countries (Bachet et al., 2020). Specifically, thymidine kinase phosphorylates FTD, which is then incorporated into DNA, leading to DNA malfunction and cytotoxicity. This mechanism of action differs from that of 5-FU and other fluoropyrimidines, which inhibit thymidylate synthase (Sunakawa et al., 2017).

The results of this real-world investigation corroborate the findings of phase III studies and worldwide recommendations, which demonstrate that FTD/TPI is a safe and effective therapy in this setting (Mulet et al., 2018; Andersen et al., 2019). Real-world, country-specific studies are significant because of the disparities in disease management that exist between regions and because of their potential to highlight treatment gaps (Ozet et al., 2022).

A systematic analysis was conducted which synthesizes published and unpublished data using FTD/TPI in clinical practice settings, comparing the outcomes of pooled analyses of observational studies, the Japanese phase II study, and the RECOURSE and TERRA phase III trials (Andersen et al., 2019). A total of 1,008 patients from 64 hospitals in Japan and Europe were compiled throughout 7 published papers between 2016 and 2018, and 2 unpublished investigations (Japanese and Danish).

Furthermore, PRECONNECT was a multicenter, open-label, phase IIIb trial, that aimed to facilitate access for eligible mCRC patients to FTD/TPI, and to further evaluate its safety and efficacy in ordinary clinical practice. 793 patients received oral FTD/TPI until disease progression, unacceptable toxicity, significant protocol deviation, physician or patient’s decision, or when the medication became commercially available (Bachet et al., 2020). PRECONNECT applied the same inclusion criteria as the RECOURSE study: adult patients with histologically proven metastatic colorectal cancer, an Eastern Cooperative Oncology Group (ECOG) performance status (PS) of 0 or 1 and at least two prior regimens of conventional chemotherapy (Mayer et al., 2015; Van Cutsem et al., 2018). Unlike RECOURSE and PRECONNECT, neither Yoshino et al. nor TERRA required patients to have had bevacizumab (or an anti-EGFR antibody for KRAS wild-type tumors) before enrollment (Yoshino et al., 2012; Xu et al., 2018; Andersen et al., 2019).

In general, the three RCTs—the Japanese phase II trial (Yoshino et al., 2012), the RECOURSE phase III trial (Mayer et al., 2015), and the TERRA phase III trial (Xu et al., 2018), the pooled analysis of observational studies (Andersen et al., 2019), the PRECONNECT trial (Bachet et al., 2020), and the present study had cohorts with baseline similar characteristics. The patients in the Romanian population were slightly older (median age of 65.5 years; patients ≥70 years represented 38% of our sample) compared to the pooled real life studies (median age of 63.5 years), Yoshino et al. (2012) and RECOURSE (median age of 63 years for both cohorts), PRECONNECT study (median age of 62 years), and TERRA trial (median age of 58 years) (Mayer et al., 2015; Xu et al., 2018; Andersen et al., 2019; Bachet et al., 2020).

Although they still accounted for more than half of the sample, male patients in the current study (52%) were relatively less prevalent than in previous investigations: Japanese phase II trial (57%), PRECONNECT population (59%), RECOURSE and the pooled observational studies (61%), and TERRA study (63%) (Yoshino et al., 2012; Mayer et al., 2015; Xu et al., 2018; Bachet et al., 2020).

Nineteen (38%) of 50 patients included in this study were RAS wild-type, whereas 19 (38%) were RAS mutant. In RECOURSE and TERRA (Mayer et al., 2015; Xu et al., 2018), mOS and mPFS were not influenced by KRAS status, whereas in the Japanese phase II study (Yoshino et al., 2012) TAS-102 was more effective in individuals with KRAS mutations. However, TAS-102 was proven effective regardless of KRAS mutational status (Yoshino et al., 2012; Andersen et al., 2019).

Overall, the median PFS for the current study was 3.85 months (95% CI: 3.1–4.6 months), higher than previously reported. A meta-analysis assessing real life experience with FTP/TPI from more than 1,000 patients reported a mPFS of 2.2 months (95% CI: 2.1–2.3 months). The randomized controlled trials had comparable median PFS: 2.0 months (95% CI: 1.9–2.8 months) for the phases II trial and TERRA study, and 2.0 months for RECOURSE (95% CI: 1.9–2.1 months). Final results from PRECONNECT study show a median PFS of 2.8 months (CI 95%: 2.7–3.0 months) (Andersen et al., 2019).

PFS is a popular endpoint that is utilized in clinical studies for third line treatment of mCRC, with radiologic testing often used as the primary basis for assessing the course of an illness. Because the true date of progression is somewhere between two radiological evaluations, using the date of scanning as the date of progression overestimates the PFS (Panageas et al., 2007). As a result, varied scanning intervals may render comparisons of median PFS across trials less relevant, as surveillance intervals may influence PFS (Andersen et al., 2019). Given the retrospective nature of this study, follow-up imaging was not subjected to the same strict requirements as a randomized clinical trial, and may account for the variations in PFS findings.

PRECONNECT study showed that the median PFS increased with duration of treatment as follows: 0–3 cycles: 2.2 (CI 95%: 2.0–2.3 months); 4-7 cycles: 5.3 (CI 95%: 4.6–5.6 months); ≥8 cycles: 9.4 (CI 95%: 8.7–10.5 months) (Bachet et al., 2020). Similarly, on the basis of univariate Cox regression analysis, our data revealed that patients who received less than five cycles of treatment had significantly inferior median PFS (3.16 months versus 7.92 months), HR = 0.18 (95% CI: 0.07–0.51), p 0.0001.

A significantly superior median PFS was observed in the case of patients benefitting from surgery (4.96, 95% CI: 3.03–6.89) compared to those who did not (2.76, 95% CI: 2.29–3.23), with log rank *p*-value < 0.0001. Surgical procedures performed in this study were either excision of the primary tumor, liver metastasectomy, palliative surgery (diverting colostomy), or debulking. No evidence connecting surgical procedures to FTP/TPI efficacy was mentioned in the literature.

Toxicities were recorded in 80% of the study population. Haematological toxicity (76%)—specifically anemia (50%), leucopenia (38%), neutropenia (34%), and thrombocytopenia (30%)—was the most prevalent adverse event, followed by fatigue (60%), and abdominal pain (18%). These results are consistent with the safety profiles of the RTCs (Yoshino et al., 2012; Mayer et al., 2015; Xu et al., 2018) and PRECONNECT study (Bachet et al., 2020).

Our study yielded results that were in line with the safety profile that had already been established for FTD/TPI, and it also showed relatively superior mPFS. This is extremely noteworthy given that both FTD/TPI and regorafenib are approved for third-line treatment in mCRC patients (Van Cutsem et al., 2018), and regorafenib is not presently covered by Romanian National Oncology Program, despite being available in several other European countries (Bullement et al., 2018). Although FTD/TPI and regorafenib have not been directly compared in a clinical study, but rather in observational series, both efficacy (Abrahao et al., 2018) and effectiveness appear comparable for mCRC patients as third line option (Masuishi et al., 2017; Moriwaki et al., 2018). A research undertaken in the United Kingdom sought to quantify the cost-effectiveness of FTD/TPI compared to other existing treatment choices for patients in this setting (best supportive care and regorafenib) from the standpoint of the National Health Service (NHS). The findings demonstrate that FTD/TPI outperforms regorafenib in terms of cost-effectiveness, with clinical outcomes much above those of patients receiving best supportive care (BSC) alone (Bullement et al., 2018).

Our study has a number of limitations. First, the research was limited by the fact that it was a retrospective, non-randomized study done at a single institution, and it only included 50 patients from a single region in Romania. Secondly, since the purpose of the study was to determine the efficacy and safety of FTD/TPI in routine clinical settings, there was no control group. Third, the lack of follow-up data required for overall survival prevented the evaluation of this endpoint. Finally, the data collection was not conducted with the same level of rigor as a randomized clinical study, and there are gaps in the information that have been provided. Real world data, despite these limitations, is crucial for consolidating clinical trial outcomes and establishing the utility of treatments among clinicians and patient subgroups.

## Conclusion

In clinical settings, new medications are often administered to a more diverse patient group in a less structured way (Andersen et al., 2019). Our study’s findings supported the safety profile for FTD/TPI that had previously been published, and demonstrated relatively superior mPFS. The results of this Romanian study support the routine use of FTD/TPI in the treatment of patients with mCRC and reflect the findings of RCTs as well as post hoc analyses conducted in other countries.

## Data Availability

The original contributions presented in the study are included in the article/Supplementary Material, further inquiries can be directed to the corresponding author.
